# Supplementary material for: Associations of SARS-CoV-2 serum IgG with occupation and demographics of military personnel
Source: PLoS One. 2021 Aug 30;16(8):e0251114. doi: 10.1371/journal.pone.0251114 (PMC8405017; doi:10.1371/journal.pone.0251114)
Supplement: S1 Fig — The % of workers (Y-axis) in different age ranges (X-axis) are depicted. Study subjects (blue bars) data from present study and local population (orange bars) estimates are from https://statisticalatlas.com/state/Connecticut/Employment-Status#figure/employment-status-by-age. (PDF) [file pone.0251114.s001.pdf]

Supplemental Material for: Associations of SARS-CoV-2 serum IgG with occupation and demographics of military personnel

Joseph Zell, Adam V Wisnewski, Jian Liu, Jon Klein, Carolina Lucas, Martin Slade, Akiko Iwasaki and Carrie A Redlich

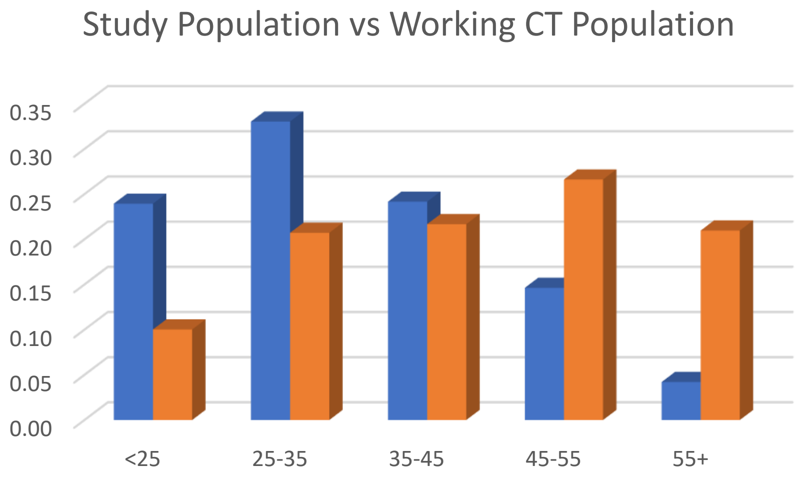

S1 Fig. Age distribution of study subjects vs. the local population. The % of workers (Y-axis) in different age ranges (X-axis) are depicted. Study subjects (blue bars) data from present study and local population (orange bars) estimates are from https://statisticalatlas.com/state/Connecticut/Employment-Status#figure/employment-status-by-age.
