# Supplementary material for: Associations of SARS-CoV-2 serum IgG with occupation and demographics of military personnel
Source: PLoS One. 2021 Aug 30;16(8):e0251114. doi: 10.1371/journal.pone.0251114 (PMC8405017; doi:10.1371/journal.pone.0251114)
Supplement: S2 Fig — The mean and standard error of spike IgG ELISA OD values are shown for subjects that belong to different MOS groups with N < 6 per MOS. (PDF) [file pone.0251114.s002.pdf]

Joseph Zell, Adam V Wisnewski, Jian Liu, Jon Klein, Carolina Lucas, Martin Slade, Akiko Iwasaki and Carrie A Redlich

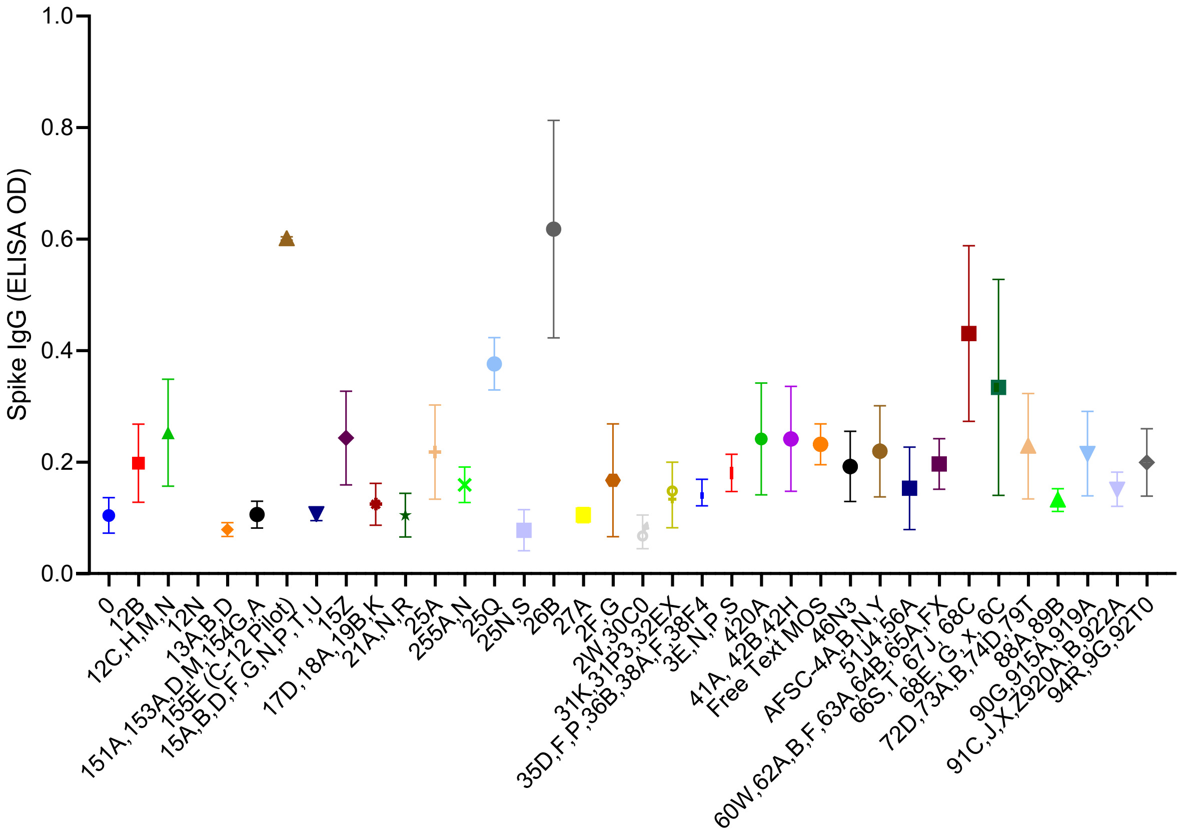

S2 Fig. Spike antigen-specific IgG in serum from subjects with different MOS codes. The mean and standard error of spike IgG ELISA OD values are shown for subjects that belong to different MOS groups with N $\leq$ 6 per MOS.
